# Construction and Evaluation of a Novel Internal Positive Control (IPC) for Detection of *Coxiella burnetii* by PCR

**DOI:** 10.5812/jjm.8849

**Published:** 2014-01-01

**Authors:** Keivan Majidzadeh, Amirhossein Mohseni, Mohammad Soleimani

**Affiliations:** 1 Tasnim Biotechnology of Research Center (TBRC), Faculty of Medicine, AJA University of Medical Sciences, Tehran, IR Iran; 2 Cancer Genetics Research Group, Breast Cancer Research Center (BCRC), ACECR, Tehran, IR Iran

**Keywords:** *Coxiella burnetii*, Molecular Detection, Q Fever, Polymerase Chain Reaction

## Abstract

**Background:**

Due to the limitations of the classical methods to detect *Coxiella burnetii*, direct diagnosis of the pathogen using PCR techniques is still the preferable approach. However, false negative results owing to the presence of PCR inhibitors are troublesome.

**Objectives:**

In order to identify the inhibitors during PCR assay, an internal positive control (IPC) was designed based on 16SrRNA gene of *C. burnetii*.

**Materials and Methods:**

In the current study, the initial and ending parts of the target gene in an external positive control plasmid (pTZ57R/T-16S) amplified using internal primers which had a *BglII* restriction site on the 5´ends. Both PCR products (fragments 1 and 2) were cloned into pTZ57R/T vector. Following *BglII* enzyme digestion, the two obtained linear plasmids were ligated. The ligation product was transformed into* Escherichia* *coli* Top10 Fʹ. Screening of the desired recombinant clone was carried out using colony PCR.

**Results:**

The size of the PCR product was equal to the sum of the first and second fragments. Sequencing confirmed the presence of the desire insert (IPC sequence) in recombinant plasmid. Consequently, the IPC fragment was longer than the target gene while both ends had similar attachments to the same primer pair.

**Conclusions:**

The results showed that direct fusion of the recombinant plasmids containing the initial and ending parts of the target gene are simple and cost-effective techniques for increasing the length of the fragment and constructing IPC.

## 1. Background

*Coxiella burnetii*, an obligate intracellular bacterium, is the causative agent of Q-fever. This bacterium has been classified as a member of center for disease control (CDC) *category B biological agents* due to its high transmission potential. Since the bacterium is an intracellular obligatory parasite, its culturing needs a live host, which is very time-wasting, expensive and dangerous and just laboratories with level 3 of biological safety and expert staff are allowed to work on contaminated samples with this bacterium. Molecular techniques such as PCR are useful methods for rapid detection of the bacterium in biological samples (1, 2). 

PCR-based technologies are rapid, sensitive, and specific for detection of infective agents that vastly developed diagnosis of a wide range of pathogens in clinical laboratories (3). However, high sensitivity of these approaches may lead to the increased number of false negative and false positive results (4). In addition to cross contamination, false positive results may be due to contamination of samples with external positive controls (5-7), whilst false negative result is caused when PCR reaction fails to work properly as a result of expiration of reaction components, technique insufficiency (wreck of equipment and facilities) or the presence of nucleic acid amplification inhibitory substances. 

Various PCR inhibitors such as EDTA, heparin, hemoglobin, ethanol and SDS may not remove during DNA extraction procedure and lead to reaction inhibition (8-14). Therefore, a strategy to provide the necessary information for accurate interpretation of PCR assay results is required. To monitor the false negative results of PCR, different standards have been developed (15). According to the guidelines of international standard organization (ISO), the presence of internal positive control (IPC) in the reaction is mandatory (16). Contrary to the external controls which are presented in a separate reaction tube, IPCs are a variety of control substances with a non-target DNA or a RNA sequence which are directly added to the PCR reaction tubes (16, 17). Internal control could determine the failure of PCR caused by inhibitor effects. In addition, the presence of internal control could ensure the accuracy of the whole nucleic acid extraction procedure (4, 16, 18, 19). Nowadays, various strategies are used to make internal positive control for diagnostic PCR assays. 

## 2. Objectives

In the present study, a unique internal positive control based on 16SrRNA of *C.* *burnetii* was constructed. 

## 3. Materials and Methods

### 3.1. Plasmid and Primer Design

The pTZ57R/T-16S plasmid was used to create an IPC fragment for PCR detection of *C. burnetii *. In an earlier study by the authors, this plasmid had been generated by cloning of diagnostic PCR product of *C. burnetii *16SrRNA (Accession number: D89799), sized 240 bp, into a pTZ57R/T plasmid and used as an external positive control ( 20 ). The 16SrRNA specific pair primers annealed to the oligonucleotides No.1-25 (forward primers; coc 1) and No. 216-240 (reverse primers; coc 4). Accordingly, an inner reverse primer (coc 2) was designed. Restriction site of *BglII *enzyme was added at 5' end of the primers. This primer annealed to the nucleotides No. 186-213 and amplified a 213 bp fragment along with coc1 primer. Also, an inner forward primer (coc 3) was designed and restriction site for *BglII *enzyme also inserted into the 5' end of this primer. This primer annealed to nucleotides No. 71-97 and amplified a 170 bp fragment along with coc 4 primer. Gene Runner (version 3.05; Hastings Software Inc., Hastings on Hudson, NY) was used for designing the primers (Table 1). 

**Table 1.** The Sequences of the Primers Used in This Study

Primer Name	Sequence ^a^	Annealing Site on Diagnostic 16S rRNA of *C*.* burnetii*
**coc 1**	5´ ATATCCTTGGGCGTTGACGTTACCC 3´	No.1-25
**coc 2**	5´AATTCAGATCTCCTCTACCATACTCAAG 3´	No. 186-213
**coc 3**	5´CAGAGAGATCTAGCTTTAATCGGAATC 3´	No. 71-97
**coc 4**	5´ATCTACGCATTTCACCGCTACACCG3´	No. 216-240

^a^ The Underlined Regions Show the *BglII* Restriction Sites

### 3.2. PCR Reaction

Two PCR reactions were set up using the designed primers. In the first reaction, primers coc 1 and coc 2 and in the second reaction, primers coc 3 and coc 4 were used. The PCR reactions were prepared according to the standard protocol in total volumes of 25 µL. In all reactions, pTZ57R/T-16S plasmid (50 ng) was used as DNA template. The reactions were run with annealing temperature at 52°C.

### 3.3. Cloning of the First and the Second Fragments

PCR products related to primers coc 1 and coc 2 (fragment one) and primers coc 3 and coc 4 (fragment two) purified using a commercial gel extraction kit (Bioneer, Korea). Ligation reaction of these fragments and pTZ57R/T vector was separately carried out by T4DNA ligase (Fermentas, Lithuania) for 3 hours at 22°C. The ligation products were transformed into *Escherichia coli* TOP10F’ and the cells were cultured onto LB agar medium containing IPTG (38.4 g/mL), X-gal (40 g/mL), ampicillin (100 g/mL) and tetracycline (50 g/mL) and incubated overnight at 37°C. The colonies receiving each of the two fragments were selected and confirmed. The plasmid of the confirmed recombinant colonies were separately extracted using AccuPrep plasmid mini extraction kit (Bioneer, Korea). Finally, the confirmed plasmids named as pTV-frg 1 and pTV-frg 2.

### 3.4. Enzymatic Digestion

Each of the plasmids pTV-frg 1 and pTV-frg 2 was separately digested and linearized using *BglII* (Fermentas, Lithuania). The digestion reaction was performed on 500 ng of each plasmid with 2 units of *BglII* in an appropriate buffer condition. Incubation was performed at 37°C for 16 hours. Finally the products were examined on 1% agarose gel.

### 3.5. Fusion of Digested pTV-frg 1 and pTV-frg 2

Since both pTV-frg 1 and pTV-frg 2 plasmids had been digested with the same enzyme, they had identical complementary sites. With regard to this competency, a ligation reaction was performed using T4DNA ligase (Fermentas, Lithuania) for 5 hours at 22°C between the two linearized plasmids. Finally, the products of the ligation reaction were transformed into the competent *E.coli* Top10F’ cells. Transformed cells were selected as mentioned above. Screening of the desired recombinant colony (containing fused pTV-frg 1 and pTV-frg 2 or IPC plasmid) carried out by colony PCR using coc 1 and coc 4 primers. Cycle sequencing method using universal M13 primers performed for final confirmation of the fused recombinant construct. The sequencing results were checked by NEB cutter’s online software V2.0 (http://tools.neb.com/NEBcutter2/) and BLAST (http://blast.ncbi.nlm.nih.gov/Blast.cgi). The confirmed recombinant plasmid containing the IPC sequence named pTZ57R/T-IPC.

### 3.6. Determination of Optimum Concentration of the IPC

To determine the optimum concentration of the IPC plasmid in PCR tubes of clinical specimens and its utility as an extraction control, the following experiment was conducted. A 10-fold serial dilutions ranging from 1 µg to 1 pg/µL of pTZ57R/T-IPC were prepared on non-infected blood samples. DNA isolation was carried out on all the IPC spiked samples by using a QIAamp® DNA Mini Kit (Qiagen Inc., Valencia, CA). The extracted DNA samples were subjected to a number of PCR reactions in the presence of coc 1, coc 4 primers and 1 ng of pTZ57R/T-16S plasmid as the target DNA (this concentration was equal to the limit of detection of the method) (20). Consequently, the minimum concentration of the IPC, that did not affect the amplification of the target gene, presented as the optimized concentration.

## 4. Results 

### 4.1. Preparation of Linear TV-frg1 and TV-frg2 Vectors

The electrophoresis of PCR products using coc 1 and coc 2 primers and coc 3 and coc 4 primers resulted in the expected products (213 bp and 170 bp, respectively) (data not shown). After cloning of both fragments and extraction of recombinant plasmids from white colonies, the presence of 213 bp and 170 bp fragments in TV-frg 1 and TV-frg 2 vectors, were confirmed using colony PCR. Enzymatic digestion on TV-frg 1 and TV-frg 2 vectors with* BglII* changed them into their linear forms. Observation of 3099 bp and also 3056 bp bands on 1% gel agarose for TV-frg 1 and TV-frg 2 vectors respectively confirmed the accuracy of digestion (data not shown).

### 4.2. Constructing IPC

**Figure 1.**
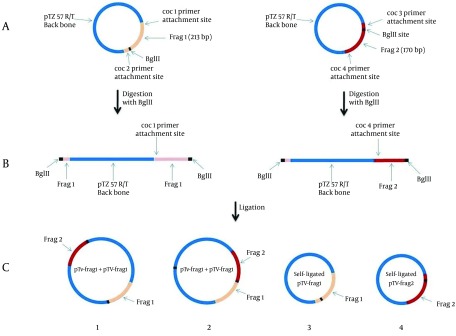
Schematic View of the Novel Strategy for IPC Construction Introduced in the Present Study A: TA-cloning of frag 1 (213 bp) and frag 2 (170 bp) corresponding to the initial and ending parts of the diagnostic 16SrRNA gene of *C*.* burnetii*. B: *BglII* digestion of pTV-frag 1 and pTV-frag 2 and creating the linearized forms. C: Ligation and cloning of both linear plasmids. As seen in the figure, the constructed plasmid No. 2 containing the IPC segment.

During the ligation process between linearized vectors pTV-frg 1 and pTV-frg 2, the creation of four arrangement states was possible (both ends of the linear vectors had *BglII *created overhangs) (Figure 1). 

**Figure 2.**
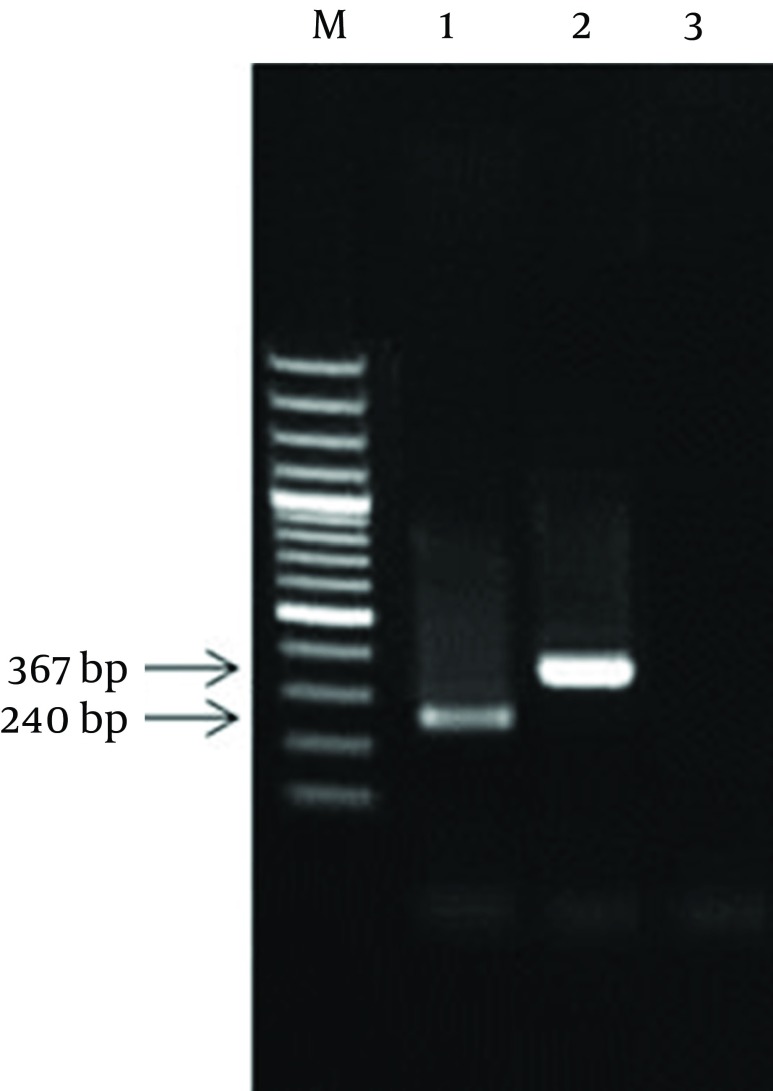
The Results of PCR of Diagnostic 16SrRNA and the IPC Sequence Using coc 1 and coc 4 Primers M: 100 bp DNA Marker; 1: PCR product of the diagnostic 16SrRNA; 2: PCR product of the IPC plasmid; 3: PCR negative control.

The colony receiving plasmid containing the complete sequence of IPC was identified by PCR using coc 1 and coc 4 primers. After IPC amplification, its PCR product showed a 367 bp band (this product was equal to the sum of the first and second fragments sizes) (Figure 2). 

Consequently, the resulted fragment (IPC fragment) was longer than the target gene while both ends had similar sticky sites to the target gene. Following sequencing of internal positive control plasmid and preparation of its restriction map, *BglII* restriction site was found in nucleotides 203 bp to 208 bp of IPC sequence, as expected. Meanwhile, the comparison of restriction maps of IPC sequence and the PCR product of 16SrRNA diagnostic gene showed complete identity between the fragment one of IPC and the initial part of the diagnostic sequence, and between fragment two of IPC and the final part of diagnostic sequence. 

### 4.3. Optimized Concentration of the IPC

**Figure 3.**
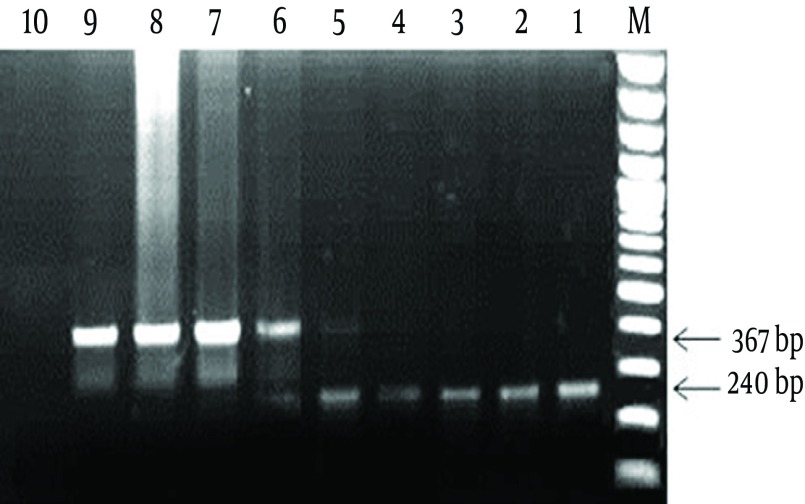
The Related Results to Optimization of the IPC Concentration M: 100 bp DNA Marker; 1: positive control for diagnostic 16SrRNA gene; 2-8: PCR products of the tubes containing 1 pg to 1 µg of pTZ57R/T-IPC plasmid in the presence of 1 ng of pTZ57R/T-16S plasmid; 9: the IPC positive control; 10: Negative control. As seen in figure, the best concentration of IPC for use in the PCR reaction was 1 ng concentration (well No. 5).

The PCR tubes containing 1 µg and 100 ng of pTZ57R/T-IPC, resulted in only amplification of IPC sequence (367 bp band). The PCR tubes containing 10 ng and 1 ng of pTZ57R/T-IPC plasmid revealed amplification in both of the IPC and diagnostic 16srRNA gene (367 bp and 240 bp). In the tubes containing 100 pg, 10 pg and 1 pg of pTZ57R/T-IPC, only amplification of the diagnostic gene (240 bp) was observed (Figure 3). Finally, 1 ng of the IPC that showed a weak 367 bp band in the presence of the lower limit of detection (1 ng) of 16SrRNA gene was determined as the optimized concentration to use in PCR reaction of clinical samples. 

## 5. Discussion

In the present study, we developed a new competitive IPC. IPCs are classified as competitive and noncompetitive. Noncompetitive IPCs include a group of internal controls with primer annealing sites different from target gene. These controls usually belong to generic controls such as universal or housekeeping genes or are manually synthesized (2, 4, 16). Murphy et al. developed a type of noncompetitive IPC; a microbiological internal positive control, for PCR based detection of *Listeria monocytogenes* and *Salmonella enterica* (4). Sohni and his colleagues, reported a microbiological noncompetitive IPC for detection of *Bacillus anthrasis* (21). Although these IPCs can take an important role in the accuracy of the results, they represent two limitations:

1. Live bacteria containing the IPCs should be apply in detection process.
2. Given the fact that two primer sets are used in a multiplex reaction, this could lead to interference of primers in the reaction and in some times could cause the formation of different primers dimers in the reaction and resulting in low sensitivity of the PCR reaction.

It is recommended to use competitive IPC instead of a noncompetitive IPC (16). Competitive IPCs have similar primer annealing sites for the target gene and are amplified by the same primer pairs and conditions along with the target gene (16, 17). In recent years, for making different competitive IPCs, several strategies have been used including altering size of target DNA by insertion, deletion, substitution or placing a new restriction site in the target DNA which is not present in the wild DNA type. Using the last strategy would add an extra step to the detection because following the PCR; the PCR product should be subjected to enzyme digestion. Occasionally, false negative results may occur due to fail of enzymatic digestions (22-28). Hodgson and his colleagues (2006) developed a competitive IPC for detection of HSV types 1 and HSV 2. They made a DNA fragment containing a primer annealing site for target gene with the heterologous region at the middle (derived from the pGEM plasmid) (22). 

One of the limitations for using the above strategies for producing IPC is that various steps and genetic engineering methods were applied, making the process difficult, complicated, time-consuming and costly whereas in the present study by a simple, easy and cost-effective strategy, an IPC fragment was developed. Since the constructed IPC was the competitive, the optimum concentration of IPC plasmid (pTZ57R/T-IPC) applicable in PCR reaction without any inhibitory effect on the amplification of the target gene was determined. The experiment showed that higher concentration of the IPC plasmid would prevent amplification of the target gene. In the current study, concerning the fact that IPC was longer than target gene in size, advantage in detection was seen because, in amplification competition between target gene and IPC, target gene is always successful.

The results of this study concluded that PCR amplification of overlapping initial and ending parts of a diagnostic gene, pasting the fragments and eventually cloning is a simple and cost-effective strategy to make longer target gene and a competitive IPC. This IPC could be routinely used for improved detection of target genes.
